# Edible aquatic Coleoptera of the world with an emphasis on Mexico

**DOI:** 10.1186/1746-4269-5-11

**Published:** 2009-04-20

**Authors:** Julieta Ramos-Elorduy, José Manuel Pino Moreno, Victor Hugo Martínez Camacho

**Affiliations:** 1 Instituto de Biología, UNAM, Apdo. Postal 70-153, 04510, México

## Abstract

Anthropoentomophagy is an ancient culinary practice wherein terrestrial and aquatic insects are eaten by humans. Of these species of insects, terrestrial insects are far more commonly used in anthropoentomophagy than aquatic insects. In this study we found that there are 22 genera and 78 species of edible aquatic beetles in the world. The family Dytiscidae hosts nine genera, Gyrinidae one, Elmidae two, Histeridae one, Hydrophilidae six, Haliplidae two and Noteridae one. Of the recorded species, 45 correspond to the family Dytiscidae, 19 to Hydrophilidae, three to Gyrinidae, four to Elmidae, two to Histeridae, four to Haliplidae and one to Noteridae. These beetles are the most prized organisms of lentic watersThe family that has the highest number of edible food insect genera and species is Dytiscidae. Here, the global geographic distribution of species in these organisms is shown, and a discussion is presented of its importance as a renewable natural resource widely used for food in various countries.

## Background

"The total volume of water in the world is about 1400 million km^3^. About 71% of the earth's surface is covered by water, with approximately 97.5% of total volume in the oceans and seas and the remaining 2.5% (35 million km^3^) in fresh water. Of this 2.5% fresh water, 2.18% is concentrated in glaciers, in the atmosphere and in underground aquifers. Because these water stores are difficult to access for use, only about 0.32% of the earth's water can be tapped. This represents 112,000 km^3^, of which 90% (100,800 km^3^) is stored as groundwater. Therefore, only a volume of 11,200 km^3 ^is available in lakes, rivers and swamps" [1].

The inland water bodies occupy a small percentage of land area. Most of these ecosystems are natural, but some are the result of human intervention, especially for generating electrical energy.

Most water bodies contain plant and animal biodiversity, including some orders of aquatic insects, that live there throughout their whole lives (beetles, bugs) or during only one part of it (for example, the larval stage of Odonata, Ephemeroptera, Trichoptera, Megaloptera, etc.). Beetles are the most abundant and diverse organisms within the Class Insecta [2]. Ratcliffe [3] states that, of the 1,750,000 species of living beings on the planet, 350,000 are beetles. This means that one in every five living beings is a beetle.

Like many other orders of insects in Mexico and throughout the world, many beetles, including some aquatic species of Coleoptera, are ingested by humans. They are also part of the food chain of different organisms.

'INSECTS AS A SOURCE OF PROTEIN IN THE FUTURE' is research area that we have developed and investigated over many years at the Institute of Biology of the National Autonomous University of Mexico (UNAM). We studied the insect species that are eaten in Mexico, where we have recorded 549 species to date [4]. Various aspects of anthropoentomophagy (ingestion of insects by humans) have been investigated in Mexico [4-23], as well as in countries throughout the world [24-26].

Anthropoentomophagic activity has also been partially documented in other countries where it is practiced, including Australia, Japan, China, Mali, Botswana United States, Canada, Peru, Colombia Venezuela and others [27-33].

According to the anthropoentomophagic literature, a taxonomic and geographic analysis of aquatic beetles that are consumed by humans has not yet been completed. This is one reason why research into the edible aquatic beetles in the world, with particular emphasis on Mexico, is of interest.

## Methods

In order to know the edible aquatic beetles in the world, bibliographic research was conducted at scientific institutions in different countries, mainly the United States (University of Wisconsin at Madison) and France (National Museum of Natural History, Museum of Man, Center of Asian Studies, Arab World Institute (IMA), etc.) as well as in Mexico in special libraries. Various topics were reviewed, including food processing, anthropological themes, anthropology of food, ethnographic, ecological, entomological, geographical, nutritional, and taxonomic studies, as well as those of historic travels of naturalists, or magazines spread and so on.

### Field work

In some cases authors reported the ethnic groups involved in the ingestion of edible insects, but others does not, so by the references of the place and country, we search the ethnos in human geography or ethnography books.

Field work was conducted in rural areas of Mexico. This included regular outings spanning four seasons, with a stay of at least fifteen days per season in several Mexican states. The aim of the fieldwork was to conduct semi-structured interviews of the ethicist type (is a technique for a person to conver orally interviewer personal definition of the situation) [34]. The inhabitants of various localities were interviewed concerning the use of water beetles in their food. Interviews were carried out in the "tianguis" (street markets), or on the streets or even in homes. The interviewees were adults of both sexes from 20 to 65 years of age. The questions were related to the "little water animals" (aquatic insects) that are used in their food, as well as how they obtain and eat them.

Sample collections were also made, and relevant comments were gathered in the "tianguis" (street markets), where samples are exhibited for sale. To collect samples, we used entomological nets or diverse equipment, in order to obtain insect specimens that are most abundant in the bodies of waters [35]. To separate samples, various tools were used, such as sieves of various diameters, forceps, vials, vacuum cleaners, brushes and tweezers. Samples could also be collected manually [36,37].

### Laboratory work

Species reported as edible were transferred to the Institute of Biology (UNAM) in Mexico City. The laboratory work included assembling, labelling, and taxonomic determination, through the use of taxonomic keys (Checklist of Coleoptera [38], Dytiscidae [39], Hydrophilidae [40], Haliplidae, Dytiscidae, Noteridae, Gyrinidae, Hydrophilidae, [41], Dytiscidae, Noteridae, Haliplidae, Gyrinidae, Hydrophylidae, Elmidae [42], Hydrophilidae [43], Edible Coleoptera of Mexico [19], Coleoptera Bibliography [44], Hydrophilidae [45], Aquatic insects [37], Dytiscidae [46]. Dytiscidae [47], Haliplidae, Dytiscidae, Gyrinidae [48], Dytiscidae [49]). Samples were catalogued in the database of Edible Insects of Mexico, at the National Collection of Insects, Laboratory of Entomology of the Institute of Biology (UNAM) [4].

## Results and discussion

### Generalities

The list of edible insects in the world [50] shows that the number of edible aquatic beetles is not very high; only 6.58% of them. However, it has been observed that in some Asian countries, such as Japan [33], China [51], Thailand Indonesia (Java and Bali) and Vietnam [52], the consumption of aquatic insects is more common. This also indicates that the use and exploitation of the ecosystem varies according to where humans settle and that people tend to use those specimens that are more readily available, more plentiful and easy to capture, store and prepare for eating.

### Collection

In order to obtain aquatic insects, people employ various types of household items. These include various kinds of nets made of aquatic plant fibers in the form of baskets of different sizes or bags made of nylon mesh. Sometimes people use various types of clothing to build a large net or buy nets directly in "tianguis" (street markets) or in specialized stores.

### Taxonomy

Seven families of edible aquatic beetles have been recorded so far; there are 22 genera and 78 species belonging to the families Dytiscidae, Gyrinidae, Elmidae, Histeridae, Hydrophilidae, Haliplidae and Noteridae. The majority of taxa have been recorded in lentic environments (including the families Hydrophilidae, Histeridae, Dytiscidae, Noteridae and Haliplidae) or in lotic environments (the family Elmidae). The family Gyrinidae is found in ponds of crystalline water or wells, where they are located at the surface.

Table 1 shows the taxonomic distribution by family, subfamily, tribe or group, genus, subgenus, species, subspecies, the author who described the species, the continent, the countries where they are eaten and the ethnic groups that consume them.

**Table 1:** Taxonomy of some aquatic edible Coleoptera of the world

**Subfamily**	**Tribe o Group**	**Genus (Subgenus)**	**Species**	**Continent**	**Country**	**Ethnos**
Family **DYTISCIDAE** Predaceous Diving beetles						
Colymbetinae	Agabini	*Gaurodytes*	*fulvipennis *(Régimbart, 1899)	Asia	China	Han
Dytiscinae	Agabini	*Platynectes*	*guttula *(Régimbart, 1899)	Asia	China	Han
Dytiscinae	Colymbetini	*Rhantus*	*atricolor *(Aubé, 1838)	America	Mexico	Otomi, Nahuatl, Mazahua, Matlazinca, Zapotec
Dytiscinae	Colymbetini	*Rhantus*	*consimilis *(Motschulky, 1859)	America	Mexico	Otomi, Nahuatl
Dytiscinae	Colymbetini	*Rhantus*	*latus *(Fairmaire, 1869)	Africa	Magadascar	Malagasy
Dytiscinae	Colymbetini	*Rhantus*	sp. (Lacordaire 1835)	America	Mexico	Nahuatl, Mixtec, Zapotec, Otomi, Yutoaztec, Mixe, Chol, Tzeltal, Tzotzil
Dytiscinae	Cybisterini	*Cybister*	*occidentalis *(Aubé, 1838)	America Africa Asia	Mexico, Cameroon, China	Mazahua, Otomi, Nahuatl, Maya, Zapotec Fulani, Hausser, Kirdi, Bantu Han
Dytiscinae	Cybisterini	*Cybister*	*frimbiolatus *(Say, 1825)	America Asia	China Mexico	Han Nahuatl, Yutoaztec, Tlapaneco, Mixtec, Mazahua, Otomi, Maya, Zapotec,
Dytiscinae	Cybisterini	*Cybister*	*tripunctatus *(Olivier, 1795)	Asia	China Japan Indonesia, Thailand	Han Nihonjin, Nipponjin Toraja, Lao
Dytiscinae	Cybisterini	*Cybister*	*hova *(Alluaud 1900),)	Africa	Madagascar Southern	Malagasy
Dytiscinae	Cybisterini	*Cybister*	*owas *(Laporte, 1835)	Africa	Madagascar	Malagasy
Dytiscinae	Cybisterini	*Cybister*	*operosus *(Sharp, 1880)	Africa	Madagascar	Malagasy
Dytiscinae	Cybisterini	*Cybister*	*bengalensis *(Aubé, 1838)	Asia	China	Han
Dytiscinae	Cybisterini	*Cybister*	*binotatus *(Boheman, 1844)	Africa	Congo	Bantu
Dytiscinae	Cybisterini	*Cybister*	*distinctus *(Régimbart, 1877)	Africa	Senegal Sierra Leone Congo	Soninke, Wolof Temne y Mende Bantu
Dytiscinae	Cybisterini	*Cybister*	*japonicus *(Sharp, 1873)	Asia	China Japan	Han, Nihonjin, Nipponjin
Dytiscinae	Cybisterini	*Cybister*	*flavocinctus *(Aubé, 1838)	Asia America	China Mexico	Han Nahuatl, Mazahua, Matlazinca,
Dytiscinae	Cybisterini	*Cybister*	*limbatus *(Fabricius, 1775)	Asia	China Laos Thailand	Han Lao, Thai, M:on-Khmer.Tibeto-Burmese.Hmong-Loumien, Lao
Dytiscinae	Cybisterini	*Cybister*	*sticticus*	Asia	China	Han
Dytiscinae	Cybisterini	*Cybister*	*lewisianus (*Sharp 1873)	Asia	China	Han
Dytiscinae	Cybisterini	*Cybister*	*guerini *(Aubé, 1838)	Asia	China Japan Indonesia	Han Nihonjin, Nipponjin Toraja
Dytiscinae	Cybisterini	*Cybister*	*sugillatus *(Erichson, 1834)	Asia	China Japan	Han Nihonjin, Nipponjin
Dytiscinae	Cybisterini	*Cybister*	*insignis *(Sharp, 1882)	Africa	Gabon	Galoas, Nkomis, Irungos
Dytiscinae	Cybisterini	*Cybister*	*singulatus*	Asia	China Japan	Han Nihonjin, Nipponjin
Dytiscinae	Cybisterini	*Cybister*	*explanatus *(Leconte, 1851)	America	North of USA and N. of Mexico	Aleut, Yupiks, Inuit, Yellowknives, Gwichin, Tanana, Dogrib, Cree, Naskapi, Montagnais Nahuatl, Cora, Huichol, Tarahumaras, Mayos, Seri, Pimes, Yaquis
Dytiscinae	Cybisterini	*Cybister (af.)*	*explanatus *(Leconte, 1851)	America	North of USA and N. of Mexico	Aleut, Yupiks, Inuit, Yellowknives, Gwichin, Tanana, Dogrib, Cree, Naskapi, Montagnais Nahuatl, Cora, Huichol, Tarahumaras, Mayos, Seri, Pimes, Yaquis
Dytiscinae	Cybisterini	*Cybister*	sp. (Curtis, 1827)	America, Asia	Mexico, Thailand, Vietnam, China	Nahuatl, Mixtec, Zapotec, Otomi, Yutoaztec, Popolaca, Huasteco, Totonaca, Tarascan, Mazahua, Maya, Lao Bana, Cham, Co-ho, Ede, Hoa, Kher, Mong, Nung, San Chay, Tày, The Thai, Han
Dytiscinae	Cybisterini	*Cybister*	*japonicus *(Sharp, 1873)	Asia	China	Han
Dytiscinae	Cybisterini	*Cybister*	*ellipticus *(Leconte, 1851)	America	USA	Appalachian, Timucua, Calus, Creek, Cherokee, Seminole, Yuchi, Catawba, Natchez, Choctaw, Chicasaw
Dytiscinae	Cybisterini	*Dytiscus*	sp. (Linneo, 1758)	Africa, Asia, America	Cameroon, China, Japan Mexico	Fulbé (Peuls), Hausser, Kirdi, Bantu Han Nihonjin, Nipponjin Nahuatl, Yutoaztec, Otomi
Dytiscinae	Cybisterini	*Dytiscus (Dytiscus)*	*marginicollis *(Le Conte, 1844)	America	Mexico	Maya, Chol, Zoque, Zapotec, Tzeltal
Dytiscinae	Cybisterini	*Dytiscus*	*validus *(Regimbart, 1883)	Asia	Japan	Nihonjin, Nipponjin
Dytiscinae	Cybisterini	*Dytiscus*	*marginalis *(Linneo, 1758)	Asia	China Japan	Han Nihonjin, Nipponjin
Dytiscinae	Cybisterini	*Dytiscus*	*habilis *(Say, 1830)	Asia America	China Japan Mexico	Han Nihonjin, Nipponjin Nahuatl, Yutoaztec, Otomi,
Dytiscinae	Cybisterini	*Dytiscus (Macrodytes)*	*circumflexus *(Fabricius, 1801)	Africa	Morocco	Arabic, Berber, Sefardi
Dytiscinae	Cybisterini	*Megadytes*	*giganteus *(Castelnau, 1834)	America	Mexico	Zoque
Dytiscinae	Cybisterini	*Megadytes*	*gigantea *(Laporte, 1834)	America	Mexico	Zoque. Mixe, Chol, Tzeltal, Tzotzil, Zapotec
Dytiscinae	Cybisterini	*Megadytes*	sp.	America	Mexico	Otomi, Otopame, Maya, Nahuatl, Zapotec, Mazahua
Dytiscinae	Eretini	*Eretes*	*sticticus *(Linneo, 1767)	Asia Africa	Myanmar Malaysia Kenya, India	Karen, Kayah, Black Karen, Padaung, Pow Karen, White Karen, Zyein, Penan/Punan Bidayuh, Melanau, Kenyah, Kayan, Kedayan, Murut, Kelabit. Bisaya, Masai, Luo, Kalefin, Kikuyus, Meu, Akamba, Gussi Parsis, Sijs
Dytiscinae	Thermonectini	*Acilius*	sp. (Leach, 1817)	Asia	China	Han
Dytiscinae	Thermonectini	*Thermonectes (Termonectes)*	sp. (Eschscholtz, 1833)	America	Mexico	Zapotec, Nahua, Otomi, Popolaca, Totonaco
Dytiscinae	Thermonectini	*Thermonectes*	*marmoratus *(Hope, 1832)	America	Mexico	Nahuatl, Yutoaztec, Totonaco, Zapotec, Huasteco, Otomi
Dytiscinae	Thermonectini	*Thermonectes*	*basilaris *(Harris, 1829)	America	Mexico	Nahuatl, Yutoaztec, Totonaco, Zapotec, Huasteco, Otomi, Popolaca, Mixtec,
Laccophinae		*Laccophilus*	*apicalis *(Sharp)	America	Mexico	Nahuatl, Otomi
Laccophinae		*Laccophilus*	sp.(Leach)	America	Mexico	Nahuatl, Yutoaztec
Family **GYRINIDAE** Girinos						
Gyrininae		?	?	Australia	Australia	Melanesia, Micronesia, Polynesia
Gyrininae		*Gyrinus*	*parcus *(Say, 1834)	America	Mexico	Tarascan, Nahuatl, Zapotec, Popolaca, Otomi, Totonaco, Huasteco, Yutoaztec.
Gyrininae		*Gyrinus (Oreogyrinus)*	*plicatus (*Regimbart, 1838)	America	Mexico	Nahuatl, Yutoaztec, Popolaca, Zapotec, Huasteco, Otomi, Totonaco.
Family **ELMIDAE** Riffle beetles						
Elminae	Elmini	*Elmis*	*chilensis *(Germain 1854)	America	Peru	Quechua, Aymara, Aguaruna, Asháninka, Machiguenga
Elminae	Ellmini	*Elmis*	*condimentaria *(Philippi, 1864)	America	Colombia Venezuela	Mestizos, Black, Amerindia, White Yeral, Yanomami, Guarao, Yaruro
		*Austrelmis = Elmis*	*chilensis *(Germain 1854),	America	Chile, Peru	Mapuches, Pehuenches, Araucanian, Aucan, Huilcaman Quechua, Aymara, Aguaruna, Asháninka, Machiguenga
		*Austrelmis = Elmis*	*condimentarius *(Philippi, 1864)	America	Chile, Peru	Mapuches, Pehuenches, Araucanian, Aucan.Huilcaman Quechua, Aymara, Aguaruna, Asháninka, Machiguenga
Family **HISTERIDAE** clown beetles or hister beetles						
		*Hololepta (Hololepta)*	*guidonis *(Marseul, 1860)	America	Mexico	Otopame, Maya, Náhuatl, Zapotec
		*Hololepta*	sp. (Paykull, 1860)	America	Mexico	Mixtec
Family **HYDROPHILIDAE** Water Scavenger Beetles						
Hydrophillinae	Hydrophillini	*Hydrophilus (Hydrous)*	*pallidipalpis *(Mac Leay, 1825)	Asia	India China Japan	Parsis, Sijs Han Nihonjin, Nipponjin
Hydrophillinae	Hydrophillini	*Hydrophilus (Hydrous)*	*bilineatus *(Mac Leay, 1825)	Asia	China Japan, Vietnam	Han Nihonjin, Nipponjin Bana, The Cham, The Co-ho, The Ede, The Hoa, Khmer, Mong, Nung, San Chay, Tày, Thai
Hydrophillinae	Hydrophillini	*Hydrophilus (Stethoxus)*	*cavisternum *(Bedel, 1891)	Asia	China (Hainan Islands) Japan Vietnam	Han Li, Zhuang, Buyei, Sui, Dong, Dai Nihonjin, Nipponjin Bana, Cham, Co-ho, Ede, The Hoa, Khmer, Mong, Nung, San Chay, Tày, Thai
Hydrophillinae	Hydrophillini	*Hydrophilus (Dytiscus)*	*hastatus *(Herbst, 1779)	Asia	China Japan Thailand Cambodia Laos Myanmar	Han Nihonjin, Nipponjin, Lao Khmer Lao, Thai, Mon-Khmer, Tibeto-Burmese, Hmong-Loumien. Karen Kayah, Black Karen, Padaung, Pwo, White Karen,
Hydrophillinae	Hydrophillini	*Hydrophilus*	*acuminatus *(Motschulsky, 1854)	Asia	China Japan	Han Nihonjin, Nipponjin
Hydrophillinae	Hydrophillini	*Hydrophilus*	*senegalensis *(Percheron, 1835)	Africa	Senegal	Soninke, Wolof
Hydrophillinae	Hydrophillini	*Hydrophilus*	*olivaceus *(Fabricius, 1781)	Asia	India	Parsis, Sijs
Hydrophillinae	Hydrophillini	*Hydrous*	*hastatus *(Herbst, 1779)	Asia	Vietnam	Bana, Cham, Co-ho, Ede, Hoa, Khmer, Mong, Nung, San Chay, Tày, Thai
Hydrophillinae	Hydrophillini	*Hydrous*	*picicornis *(Chevrolat, 1863)	Asia	Philippines	Aeta, Iloko, Austronesian, Visayas, Tagalog, Manobo, Negrito.
Hydrophillinae	Hydrophillini	*Hydrous*	sp.	Asia	Thailand	, Lao
Hydrophillinae	Hydrophillini	*Hydrous (Tempnopterus)*	*Marginatus*	Africa	Senegal	Soninke, Wolof
Hydrophillinae	Hydrophillini	*Tropisternus*	sp (Solier 1834)	Amèrica	Mexico	Nahuatl, Mixtec, Zapotec, Otomi, Yutoaztec
Hydrophillinae	Hydrophillini	*Tropisternus (Hydrophilus)*	*collaris *(Fabricius, 1775)	Asia	Japan	Nihonjin, Nipponjin
Hydrophillinae	Hydrophillini	*Tropisternus (Tropisternus)*	*mexicanus *(Castelnau, 1840)	America	Mexico Panama SudAmerica	Nahuatl, Zapotec, Yutoaztec Ngobe, Kunas, Wounan, Bribris
Hydrophillinae	Hydrophillini	*Tropisternus*	*tinctus *(Sharp, 1882)	America	Mexico	Nahuatl, Mixtec, Zapotec, Otomi, Yutoaztec, Maya,
Hydrophillinae	Hydrophillini	*Tropisternus*	*sublaevis *(Leconte, 1855)	America	Mexico	Nahuatl, Yutoaztec, Otomi, Zapotec, Mixtec
		*Berosus*	sp. (Leach, 1817)	America	Mexico	Nahuatl, Yutoaztec
		*Diloboderus*	sp.(Reiche 1859)	America	Mexico	Tzeltal, Maya, Tojolabal, Zapotec, Zoque
		*Dibolocelus*	sp. (Regimbart, 1901)	America	Mexico	Huichol, Cora, Tepehua
Family **HALIPLIDAE** Crawling water beetles						
		*Haliplus*	*punctatus *(Aubé, 1838)	America	Mexico	Nahuatl, Yutoaztec, Popolaca, Zapotec, Huasteco, Otomi, Totonaco
		*Haliplus*	sp. (Latreille 1802)	America	Mexico	Tlapaneco, Nahuatl, Mixtec, Amuzgo
		*Peltodytes*	*mexicanus (*Wehncke 1883)	America	Mexico	Nahuatl, Yutoaztec, Otomí, Zapotec, Mixtec
		*Peltodytes*	*ovalis *(Zimmerman 1924)	America	Mexico	Nahuatl, Mixtec, Zapotec, Otomí, Yutoaztec
Family **NOTERIDAE** Burrowing water beetles						
		*Suphisellus*	sp.	America	Mexico	Nahuatl, Yutoaztec Huasteco, Tarascan, Otomi

Aquatic insects in the wild live mainly in lentic waters of ponds, lakes, rivers and small streams, as well as puddles, "jaguey" (pool), wetlands or dams. Table 2 identifies the habitats in which some families of aquatic beetles are located.

**Table 2:** Habitats of some families of edible aquatic Coleoptera

Families	Habitats
Dytiscidae	Lakes, streams, creeks, rivers, springs and fountains.
Elmidae	Lakes, streams, creeks, rivers, springs and fountains.
Gyrinidae	Puddles natural or artificial ponds, stagnant water from rivers, streams, creeks.
Haliplidae	Streams, creeks, rivers
Hydrophilidae	Brooks, streams, rivers, marshes, swamps, springs, springs, the lake, the rivers and beaches.
Histeridae	Underwater and Coastal.
Noteridae	Pools, ponds and lakes covered with weeds

* [53], + [37]

**Figure 1:**
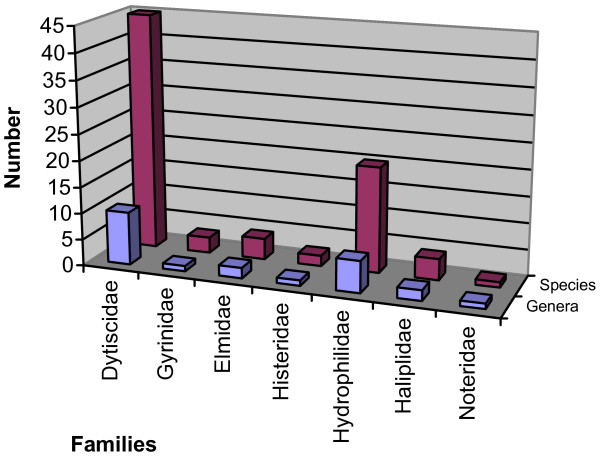
**Genera and Species Number of Edible Species of Aquatic Coleoptera of the World**.

Figure 1 shows the variation in the number of families (7), genera (22) and the species (78) that belong to each of them. In relation to species number, the family Dytiscidae includes 57.69%, Gyrinidae 3.84%, Elmidae 5.12%, Histeridae 2.56%, Hydrophilidae 24.35%, Haliplidae 5.12% and Noteridae 1.28%. The families best represented by species number are Dytiscidae (45), followed by Hydrophilidae, which has six genera and 19 species. This is likely due to the wide geographic distribution of these families, which occur in various types of waters and ponds.

The family Dytiscidae contains 40.91%, Gyrinidae 4.55%, Elmidae 9.09%, Histeridae 4.55%, Hydrophilidae 27.27%, Haliplidae 9.09% and Noteridae 4.55%.

The percentage of edible species (78), which correspond to the families Dytiscidae (57.69%), Hydrophilidae (24.36%), Gyrinidae (3.85%), Haliplidae (5.13%), Elmidae (5.13%), Histeridae (2. 56%) and Noteridae (1. 28%).

### Biogeography

**Figure 2:**
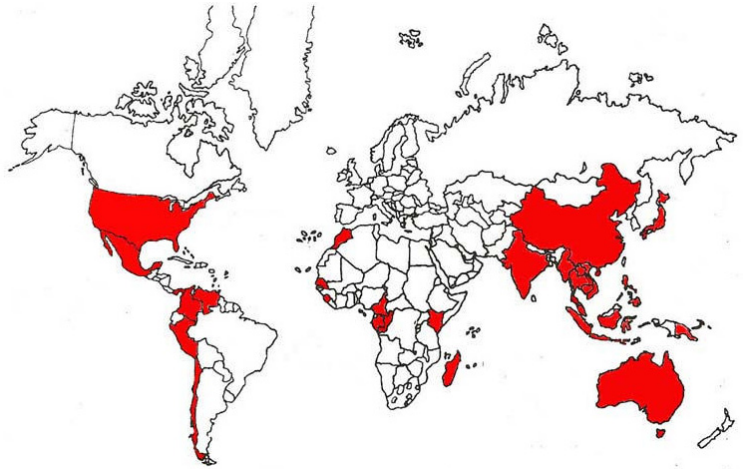
**Ingestion of Aquatic Coleoptera in the World**.

The genera *Cybister, Dytiscus, Hydrophilus, Elmis, Hydrous *and *Tropisternus *are consumed in several countries. The practice of eating aquatic beetles has been reported in various countries throughout the world We report here that the consumption of aquatic insects is practiced in 27 countries worldwide (Figure 2) and occurs in some developed countries (Australia, U.S.A., Japan for example), as well as in many underdeveloped countries (Mexico, Malaysia, Gabon, Cameroon etc.).

This indicates that there is a convergence in eating habits of various ethnic groups that take advantage of aquatic ecosystems. However, it can also be seen that a systematic investigation of edible aquatic beetles in many other countries has not yet made.

**Figure 3:**
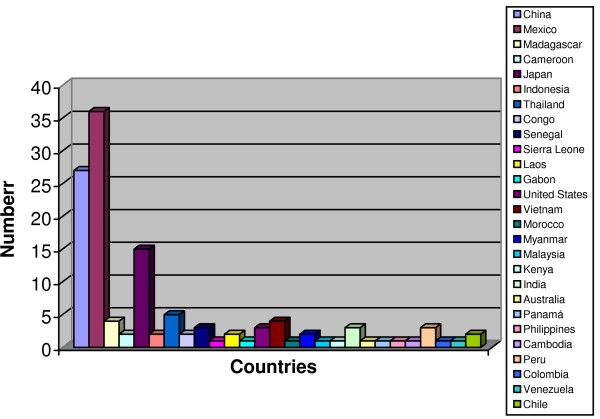
**Aquatic Coleopterophagic Countries of the World**.

Figure 3 shows the countries where the consumption of edible aquatic beetles is practiced. Some countries use many species. For example, 36 species are used in Mexico, 26 in China and 15 in Japan. The highest number of recorded species is reported in Mexico. One might suspect that this is the most anthropoentomophagic country, but, in reality, it is because Mexicans have long been systematically researching the use of insects as food.

### Interviews and tracking

Interviews conducted in different localities in Mexico revealed a high consumption of a large variety of aquatic organisms. These are located in several different inland water bodies, depending on the ecological characteristics of the geographic regions where they are found. These insects were found during all seasons; however, people living near bodies of water reported that insects have a season of natural abundance. For example, *Dytiscus *is abundant in the months of February, March and April in warm regions. Insects in general are more abundant during the rainy season, when habitats are richer in organic matter and are larger. This is the case for the larvae of dragonflies, known as "padrecitos" (Odonata: Aeschnidae, Coenagrionidae, Libellulidae) and for Mayflies, the May month (Ephemeroptera: Ephemeridae, Baetidae, Leptophlebiidae). In seasons when the waters are "tame" and have little flow, other insects are abundant, such as "manfes" (Megaloptera: Corydalidae) and the "cargapalitos" (Trichoptera: Hydropsychidae, Leptoceriidae, Rhyacophilidae).

### Collecting and gender roles

In general, edible aquatic insects are collected at random by women, children or men. They use baskets, cloth nets or collect manually. The people are very familiar with the different " l edible little animals" (animalitos) and their various common names.

### Preparation

Aquatic beetles are highly prized in the kitchen. They are prepared roasted or smoked and are used in "tamales", "quesadillas", "sopes", etc. Either they are boiled in salt water and then combined with pepper and lemon, or they are dried in the sun or in the "comal". (is a traditional cookware, a cast iron plate)Insects can be fried or prepared by steaming; some people also eat them alive, as *Dytiscus *of Epatlan Lagoon of Puebla State.

In some fine restaurants, insects are prepared in various ways according to the ingenuity and creativity of chefs and therefore have been transformed into gourmet dishes. In addition, people have said that the taste of the beetles is varied; they reported their similarity to octopus, shrimp powder, fish, or crabs, and they sometimes explain that the beetles, in general, taste and smell like seafood.

### Sales

**Figure 4:**
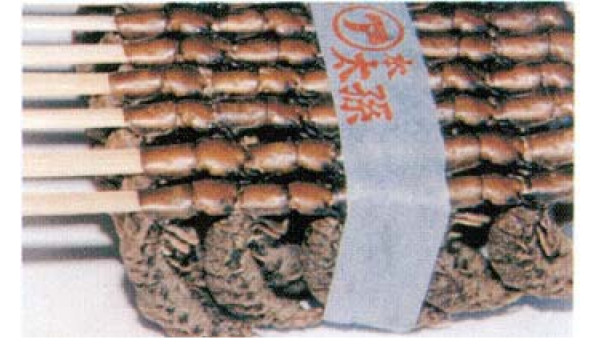
**Larvae of Megaloptera boiled in salt water and ready to eat in skewers sold at supermarkets in Japan (photography of Dr. Jun Mitsuhashi**.

These insects are mostly consumed in rural areas by the families involved in their collection and preparation, in addition being consumed by people from other social strata. During their local season, insects are sold in "tianguis" (local informal markets of villages and towns) and formal markets, including some in city of Mexico. In Japan larvae of Megaloptera species are sold in packages of 12 skewers (Figure 4). Insects are bought by people of various economic levels. In addition, some middlemen or restaurant owners have received the largest gains without making any effort to obtain them. Thus, edible aquatic insects are widely known, and the species most in demand are consumed and marketed in both rural populations as well as in semi-urban and urban populations.

They are sold by package, by sample if the species is large as *Dytiscus *or *Cybister*. In Mexico, it costs from $1 to $3.5 USD by individual, depending on the locality, or sold by measure if they are samples of little size. The cost of a sardine can of aquatic insects oscillates from $4 to $5.5 USD. of *Abedus dilatatus *(Say) in Mexico.

## Discussion

Throughout the year and particularly during the rainy seasons, there is an abundance of aquatic beetles in various inland water bodies. People of many ethnic origins (153 recorded) living at many latitudes in various countries throughout the world captured those beetles species located in puddles, ponds, pools, lakes, rivers, etc.. Aquatic beetles are consumed in both immature and adult stages.

The number of recorded species of aquatic beetles are 78, lower than that recorded for the world's terrestrial Coleoptera 499 species[54].

Table 3 shows the relationship of families and the number of species represented in Mexico (36), compared with those reported for the world. In Mexico, there is a large diversity of edible insects (547 species recorded to date). From the 126 species (23.03%) of Mexican edible coleopterans reported 36 are aquatic [19].

**Table 3:** Comparative Number of Species of Aquatic Edible Coleoptera

	**Mexico**	**World**
Dytiscidae	20	45
Hydrophilidae	7	19
Haliplidae	4	4
Gyrinidae	2	3
Histeridae	2	2
Noteridae	1	1
Elmidae	-------	4
Total	36	78

Globally, 78 species of aquatic coleopterans are consumed – that is, a little more than double what is consumed in Mexico. Both in Mexico and worldwide, the families best represented (and thus most consumed) are Dytiscidae and Hydrophilidae.

The habit of eating aquatic insects is very common today in many parts of the world, particularly in countries within Africa, Asia and America and even Australia. This assertion is in agreement with a number of reports issued by the Organization of the United Nations for Food and Agriculture (FAO). In most regions where insects are used for food purposes, species of beetles are the most common, forming a regular part of the diet. , as the sago grub (*Rhynchophorus ferrugineus *(Olivier) or *Rhynchophorus bilineatus (*Montrouzier)) in Southeast Asia and Melanesia. Also the American, African, Asian and Australian *Rhynchophorus *species, or the little coconut larvae of the beetle *Pachymerus nucleorum *(Fabricius) of the tropical regions of America as Brazil, or the big one Xylotrupes mniszechi tonkinensis (Minck.) a traditional edible beetle in Thailand and other Asian countries eaten as larvae, pupae, adults, of *Rhynchophoprus *spp are all eaten in immature and adult stages, as is happens with *Megasoma elephas (*F.) in all America, and *Dynastes hercules *L. in Brazil, Colombia, Bolivia y Mexico etc. In reference to aquatic beetles, many species of *Dytiscus *genus are cooked in different ways (roasted, in soup, grinded in sauces, mixed with eggs or with legumes in different salads, etc,) or only boiled in many countries all over the World, as well as, *Cybister hova *(Alluaud) in Madagascar, *C. japonicus *(Sharp) in Japan, *C. explanatus *(Le Conte) in all America, *Hydrous hastatus *(Herbst) in all Asia or *Tropisternum tinctus *(Sharp) and *Gyrinus parcus (*Say) in Mexico, eaten as a traditional nutritive, abundant and for free food principally for rural people.

In addition to beetles nutritional value, [12,13,15,20,22], some economists have investigated the potential for edible insects to provide income and generate jobs for the rural population [56]. This income could be provided by capturing and preparing edible insects or even raising them as "protocultures" (different kinds of care given by people in rural areas to some insect species, in order to avoid falling stocks by predation, parasitism or lack of food as well as changes in temperature, for example, increasing the organic matter content in the water where beetles and other aquatic insects are present) or doing formal cultures, which also then could be transported to urban or semi-urban areas to sell.

For the above reasons, edible aquatic beetles play an important role in the nutrition and economy of rural people [5]. They are highly prized and are also subject to national or international trade.

International trade in edible insects is important in African countries like Sudan and Nigeria that export edible insects to France and Belgium. According to the FAO, these two countries import about 5 and 3 tons, respectively, of a type of dried caterpillars from the Democratic Republic of Congo. For example, the annual exports of these caterpillars to Belgium have a value of $ 41,500 US dollars.

This marketing opportunity can be increased through the management of insect biology, establishment of protocultures and rustic cultures in industrial or artisanal quantities, or through the promotion and selection of methods and techniques of food technology. In this way, perhaps insect cultivation can be transformed into a profitable agroindustry such as that of silk or honey bee.

For the reasons explained above, gathering of edible insects is a good source of income in Japan, China, Mexico and the Congo. In addition, its exploitation requires little investment.

## Conclusion

Edible insects are generating hard currency for countries that operate locally or internationally. For South Africa, Van der Waal [56] reports that the sale of grasshoppers is a million dollar business and also in other countries[55].

As we have seen, insects are eaten in various countries. In this case China, Mexico and Japan are the largest consumers, and in China, there is cultivation of some species.

## Competing interests

The authors declare that they have no competing interests.

## Authors' information

Dra. Julieta Ramos-Elorduy: has the highest position as researcher at the Institute of Biology of the National University of Mexico and professor of postgraduate courses at the Faculty of Science of the same University. She have 104 scientific publications and four books published. 1153 cites of its publications and 1316 on internet. She lead 152 thesis and publish 289 divulgation articles.

M.en C. José Manuel Pino Moreno: Biólogist and M.Sc. by the Faculty of Science of the UNAM (National University of Mexico), Academic Technic of the Institute of Biology and Professor of the Faculty of Sciences both of the UNAM. He has published like co-author several articles about antropoentomophagy and medicinal insects and 1 book.

Biologist Victor Hugo Martínez Camacho by the Faculty of Science of the UNAM (National University of Mexico), he has published like co-author one chapter of book and several articles of edible insects.
